# China’s health assistance to Africa: opportunism or altruism?

**DOI:** 10.1186/s12992-016-0217-1

**Published:** 2016-12-03

**Authors:** Shuang Lin, Liangmin Gao, Melissa Reyes, Feng Cheng, Joan Kaufman, Wafaa M. El-Sadr

**Affiliations:** 1 School of International and Public Affairs, Columbia University, New York, USA; 2 School of Social Sciences, Tsinghua University, Beijing, People’s Republic of China; 3 Mailman School of Public Health, Columbia University, New York, USA; 4 Research Center for Public Health, Tsinghua University, Beijing, People’s Republic of China; 5 Center for Global Health and Infectious Diseases, Tsinghua University School of Medicine, Beijing, People’s Republic of China; 6 Schwarzman Scholars Program, Stephen A. Schwarzman Education Foundation, New York, USA; 7 Global Health and Social Medicine, Harvard Medical School, Boston, USA; 8 ICAP at Columbia University, New York, USA

**Keywords:** China-Africa health aid, Health diplomacy, Public health, Foreign assistance, Development aid

## Abstract

China has made substantial health commitments to Africa in the past several decades. However, while much has been written regarding China-Africa aid overall, relatively little attention has been given to China’s health aid. To better understand these investments, we provide an overview of the current framework and characteristics of China’s health aid to Africa. China’s health assistance has been perceived by some as opportunistic, largely as a demonstration of China’s engagement in “soft power” and an attempt to enhance its access to natural resources and political favors by African countries. Others have attributed altruistic intent, aiming to support the advancement of the health of populations in the African continent with a “no strings attached” approach. Our overview demonstrated that despite the magnitude of China’s health assistance, many questions remain regarding the scope of this aid, its effectiveness and the governance mechanisms that guide the conceptualization and implementation of such efforts. We also identified the need for a systematic and rigorous evaluation of the various elements of China’s health assistance to African countries in order to gain a deeper understanding of how priorities and allocations for health aid are determined, how such aid fits within the specific African country’s health strategies and to assess the effectiveness of such aid. Insights garnered through such an assessment could help determine future priorities for investment as well as inform efforts to optimize the value of China's aid for the populations of the recipient countries.

## Background

By the end of 2009, African and Asian countries had received about 80% of China’s 256 billion RMB (37.5 billion USD) in cumulative foreign aid, and in 2009 aid to Africa made up 45.7% of China’s total aid [1]. As part of this investment, health-related aid is a key priority, with China now ranking among the top 10 bilateral global health donors to Africa [2]. In this paper, we provide an overview of China’s health aid to gain a deeper understanding of the driving force behind this aid, its magnitude and the type of aid provided.

Overall, it is estimated that at least 3 billion USD from 2000 to 2012 has been committed by China to about 255 projects in health, population and water and sanitation sectors in Africa [2]. The most typical form of China’s health assistance has been the dispatching of Chinese medical teams (CMT). In 2014 for example, there were 43 CMT sent to 42 African countries [3]. The CMT program operates at an estimated annual cost of 200 million to 409.6 million RMB (29.45 million to 60 million USD) [4, 5]. Apart from the CMT program, China also has assisted in the construction of health facilities, the contribution of medical equipment and drugs, and the training of African health care workers. By 2014, China had helped build 30 hospitals and 30 malaria prevention and control centers in Africa, invested 800 million RMB (123.9 million USD) in medical equipment, supplies and anti-malaria drugs, and trained over 3,000 health care workers from various African countries [3].

### Policy and mechanisms

From its very beginnings, China’s health assistance has been perceived as opportunistic by some while at the same time as altruistic by others. Chinese overseas health assistance has its roots in the ideological concerns during the Cold War when China dispatched its first medical team to Algeria in 1963 [6]. Since that time, China has indicated that its aid was grounded in the “Eight Principles for Economic Aid and Technical Assistance to Other Countries” originally articulated by Premier Zhou Enlai in 1964, which were described as anchored in “equality, mutual benefit with no strings attached” [1, 7]. China’s current assistance and investment policies in Africa are largely guided by the 2006 document entitled, “China’s African Policy”, which indicates that China “will continue to send medical teams and provide medicines and medical materials to African countries, and help them establish and improve medical facilities and train medical personnel.” It also states that “China will increase its exchanges and cooperation with African countries in the prevention and treatment of infectious diseases including HIV/AIDS and malaria and other diseases, research and application of traditional medicine and experience concerning mechanism for public health emergencies” [8].

The 2006 document “China’s African Policy” granted the Forum on China-Africa Cooperation (FOCAC) a central role in shaping the China-Africa relationship. At each FOCAC summit since 2000, the Chinese government makes new assistance commitments, often including health-related aid. For example, at the Second Summit of the FOCAC in December 2015, Chinese President Xi Jinping announced a 60 billion USD package to support cooperation with Africa for the following three years, with public health being one of the areas to be covered [9].

Through FOCAC programs, China has established a series of other China-Africa health-related cooperation instruments. These include: the African Human Resources Development Fund in 2000; the first China-Africa Forum on Traditional Medicine in 2002; a 5 billion USD China-Africa Development Fund in 2006; and the first Ministerial Forum on China-Africa Health Development in 2013, which was timed to commemorate the 50^th^ anniversary of the first CMT sent to Algeria in 1963.

Despite these many initiatives, a key limitation of China’s health aid approach is the absence of a cohesive approach and a coherent strategy of China’s health diplomacy. It should be noted that China’s health aid is primarily managed by the Ministry of Commerce, with the participation of the Ministry of Foreign Affairs, the Ministry of Finance and the National Health and Family Planning Commission, with oversight from the State Council [10]. While the Chinese national government takes the lead to initiate and negotiate bilateral agreements with African countries, the implementation of these agreements rests with China’s provincial or subnational governments. In addition, China’s existing foreign aid policies are based largely on *ad hoc* central ministerial documents and regulations, which are not subject to approval by the legislative branch [11]. Furthermore, competition within the Chinese government ministries and at provincial levels further undermines the overall cohesion and coordination of China’s aid [12]. For example, individual provincial Chinese governments are designated to provide specific African countries with medical teams, thus, this results in the allocation of different levels of resources to health projects based on provincial interest and their local capacity [13]. This has resulted in variations in the quantity and quality of the assistance provided to recipient African countries [14].

### Measuring the magnitude and impact of China’s health assistance to Africa

One of the major challenges in understanding the scope and impact of China’s health assistance to Africa is the lack of information regarding the amount of such aid (or its overall foreign aid), the criteria for making aid decisions, and the mechanism for determining the impact of aid [15]. For example, Li found that it was difficult to find a consistent report of even the total number of CMTs dispatched to Africa [16]. Some have argued that the lack of transparency reflects China’s effort to avoid pressure both from African governments for more aid and from domestic criticism against supporting other countries while poverty remains a challenge at home [17]. Others pointed out that recipient African countries are also unwilling or incapable of informing other states on the amount of aid received from China [18].

AidData, a partnership between the College of William and Mary, Brigham Young University and Development Gateway, has attempted to track China’s foreign aid. AidData adopted a “Tracking Under-Reported Financial Flows (TUFF)” methodology [19] to collect comprehensive and standardized data on development finance flows between China and African countries. However, others have noted varying estimates of such aid by using different methods. Grepin et al., using data released from AidData, suggested that China had pledged health projects valued at 231 million USD per year over the past decade [2]. In contrast, using manually collected information, Liu et al. [5], estimated that China disbursed 150 million USD in health aid annually.

### China’s approach to aid: altruism or opportunism

China’s engagement in Africa health aid has differed from aid provided by traditional Western donors, particularly in the way China builds on its own prior experiences in building its own health system as a developing country, and in the manner in which it places special emphasis on the issue of the national sovereignty of recipient countries.

A distinctive feature of China’s health aid that is frequently discussed in the literature is what has been referred to as the “no strings attached” altruistic approach which “never imposes ideology, values and development models on other countries, especially African countries” [20]. Concerns have been raised regarding this claim indicating that the vision of a democratic Africa where human rights are respected is challenged by China’s approach, particularly with its growing influence in the continent [21]. Some have noted that China legitimizes and encourages Africa’s most repressive regimes, which leads to enabling weak and failed states [13]. Critics have also expressed concern that despite its stated approach of “no strings attached”, China has its own expectations from recipient countries, including diplomatic loyalty on issues such as Taiwan, Tibet and the status of the Uyghur people [15].

Other observers claim that China’s health priorities in Africa aim to secure stable supplies of oil and other natural resources [22]. They point to the fact that China imports one-third of its oil from Africa and significant amounts of minerals and other raw materials, largely from Sudan, Angola, and the Democratic Republic of the Congo [23].

The Chinese government has responded to some of the criticisms cited above by denying that it has any intent of plundering Africa’s resources or that its sole interest rests on utilizing Africa’s markets for its products [24]. As evidence to the support, some have noted that China provides aid to all countries in sub Saharan Africa (except to those that do not follow the “One China Policy”, i.e. that recognize Taiwan). This has been contrasted by Chinese officials with the approach by traditional Western donors, where some countries are favored over others [25]. In addition, studies have indicated the lack of evidence to suggest that China targets health aid preferentially to resource-rich countries [2]. It is further argued that China’s Official Development Assistance (ODA) to Africa has been driven primarily by foreign policy considerations instead of economic interests [26].

China has also cited as evidence of its altruistic intent the fact that African governments have expressed gratitude for China’s medical teams lauding their strong commitment and their willingness to help the citizens of their countries [27]. It is estimated that by 2009, more than 21,000 Chinese medical workers have provided services to 260 million patients around the world [1]. In addition, as of 2013, about 1000 Chinese medical team members have received medals of appreciation from recipient governments [28]. Their efforts have been acknowledged as filling critical gaps, particularly through their service in rural areas, positions that have been difficult to fill with African physicians [29]. In addition, some have countered the criticism to China by indicating that the western approach to aid, that is often couched in altruism, has failed to take the African reality into consideration and reflecting an arrogant premise of “knowing what is best for Africa” [30].

### Health aid as a diplomatic strategy

While policy dynamics behind China’s health aid attract relatively little attention from scholars, some studies have pointed out that China’s health aid has been an integral part of the country’s diplomatic strategy to expand international influence while improving its international image [6, 11]. A review [31] of China’s health diplomacy in Africa showed that in 1950s and 1960s, China’s relationship with African countries was framed as a counterbalance to the powers of United States and the Soviet Union. In the ensuing decades, China paid less attention to Africa as it sought to find its place in the international marketplace. In recent years, Africa has regained high prominence in China’s overall foreign policy, and the Chinese government has reinvigorated its commitment to health assistance for African countries as a means to strengthen its diplomatic relations.

It should be noted that China’s medical cooperation in Africa has corresponded to China’s diplomatic strategy [16]. For example, China began sending medical teams to Senegal in 1975, but withdrew such teams from 1996 to 2007, a period when diplomatic relations were severed. In addition, Thompson and Youde posited that providing healthcare resources not only helps China gain favorable trading terms and access to necessary resources, but also supports the government’s attempts to portray itself as a good international citizen [31, 32]. However, despite the fact that China has become more active in global health, Huang warns that China’s pursuit of health as primarily foreign policy could undermine its incentives and efficacy for greater international health engagement [6].

### Way forward

As China has emerged as a major global power, its interest in Africa has been described in four dimensions: political, economic, security and ideological [12]. Consequently, several authors have highlighted the importance for China to make concerted efforts to enhance its image in response to the many critiques that it faces [33]. In addition to the perception of China as acting in an opportunistic manner, concerns have also been raised as to whether China has the willingness and capacity to sustain and expand its health commitment to Africa. For example, China’s health assistance to foreign countries may be hindered by domestic pressure to provide healthcare for its own people [11, 31]. Also, with the increasingly profit-based character of China’s health system, many Chinese physicians may not be willing to serve overseas as part of medical teams, especially physicians from economically developed Chinese provinces [34, 35].

Perhaps, one strategy that may advance China’s standing as a health assistance donor is for China to become more directly involved in the global health dialogue and to commit to a careful evaluation of its health assistance investments. In recent years, a trend can be observed in which China has adjusted its health aid focus towards a more diversified approach that aims to contribute to the development of local health systems [36]. This shift may have been motivated by the outbreak of severe acute respiratory syndrome (SARS) in China in 2002, which prompted China’s recognition of the importance of public health not only as a domestic issue but also as global public good [37]. China, along with other BRIC countries, has seen strong growth in its total health expenditure while at the same time its contribution to the global health care market is believed to have surpassed those of most G7 economies [38]. The recent Ebola outbreak in West Africa revealed the fragility of health systems in the affected countries and to some extent reinforced for China the need to engage in strengthening health systems. Consequently, China was one of the first countries to provide aid and medical workers to the region at the height of the Ebola crisis [39]. Similarly, China is also participating in an international task force to design the African Centers for Disease Control and Prevention (African CDC), with an additional 2 million USD contribution to its 2016 budget [40]. China has also affirmed its commitment to universal health coverage (UHC) and is working with African governments to build their own capacity to achieve UHC, focusing on technology transfers, pharmaceutical capacity around essential medicines and training of African health personnel [39, 41]. These new efforts suggest an encouraging new direction for China with the demonstration of a more explicit commitment to public health and sustainable development rather than its traditional focus on curative medicine and “brick and mortar” infrastructure.

## Conclusions

In summary, many decades after China began its health assistance efforts to support African countries, there is a paucity of information regarding its scale, scope and impact. Irrespective of the nature of the assistance, there is the need for a systematic and rigorous evaluation of the various approaches and investments. Such assessments could be critical in informing future investments and consequently serving to advance the health of the target populations.

In an era characterized by a plateau in the amount of external assistance for health to resource-limited countries, it is critically important that China's funding is effectively and efficiently utilized. China has become a major contributor of health assistance in Africa. If China’s investments in Africa can be optimally conceptualized and implemented, such resources can substantially contribute to the health and well-being of those in most need in the African continent.

## Abbreviations

BRIC: Britain, Russia, India and China
CDC: Centers for Disease Control and Prevention
CMT: Chinese medical teams
FOCAC: Forum on China-Africa Cooperation
G7: Group of seven countries, an informal bloc of industrialized democracies--the United States, Canada, France, Germany, Italy, Japan, United Kingdom
ODA: Official Development Assistance
RMB: Chinese Yuan Renminbi (official currency of China)
SARS: Severe acute respiratory syndrome
TUFF: Tracking under-reported financial flows
UHC: Universal health coverage
USD: United States dollar
