# Correction to: China’s health assistance to Africa: opportunism or altruism?

**DOI:** 10.1186/s12992-018-0411-4

**Published:** 2018-10-01

**Authors:** Shuang Lin, Liangmin Gao, Melissa Reyes, Feng Cheng, Joan Kaufman, Wafaa M. El-Sadr

**Affiliations:** 1 School of International and Public Affairs, Columbia University, New York, USA; 2 School of Social Sciences, Tsinghua University, Beijing, People’s Republic of China; 3 Mailman School of Public Health, Columbia University, New York, USA; 4 Research Center for Public Health, Tsinghua University, Beijing, People’s Republic of China; 5 Center for Global Health and Infectious Diseases, Tsinghua University School of Medicine, Beijing, People’s Republic of China; 6 Schwarzman Scholars Program, Stephen A. Schwarzman Education Foundation, New York, USA; 7 Global Health and Social Medicine, Harvard Medical School, Boston, USA; 8 ICAP at Columbia University, New York, USA

## Correction

Please note that following publication of the original article [1], one of the authors has flagged that the abbreviations section lists “BRIC” as “Britain, Russia, India and China”.

However, in accordance with reference ‘38’ [2], in relation to which ‘BRIC’ is referred, the correct meaning of BRIC is in fact: **“Brazil, Russia, India and China”.**
